# Use of Pleuroperitoneal Shunt in Chylothorax Related to Central Line Associated Thrombosis in Sickle Cell Disease

**DOI:** 10.3390/children5010007

**Published:** 2018-01-02

**Authors:** Elizabeth Spiwak, Chad Wiesenauer, Arun Panigrahi, Ashok Raj

**Affiliations:** 1 Department of Pediatrics, University of Louisecille, Louisville, KY 40202, USA; 2 Department of Pediatric Surgery, University of Louisville, Louisville, KY 40202, USA; chad.wiesenauer@louisville.edu; 3 Department of Pediatric Hematology-Oncology, UC Davis, University of California, Sacramento, CA 95817, USA; arpanigrahi@ucdavis.edu; 4 Department of Pediatric Hematology-Oncology, University of Louisville, Louisville, KY 40206, USA; ashok.raj@louisville.edu

**Keywords:** chylothorax, sickle cell disease, central vein thrombosis, pleuroperitoneal shunt

## Abstract

Central vein thrombosis as a cause of chylothorax is uncommon, and in a few cases in the literature was related to thrombotic complications of central venous access devices (CVAD). Superior vena cava (SVC) occlusion-induced chylothorax has been described in adult sickle cell disease (SCD) in a setting of chronic indwelling CVAD. There are limited reports on chylothorax induced by central venous thrombosis secondary to chronic CVAD in children with SCD. We describe an 8-year-old male patient, with a history of SCD, maintained on long term erythrocytapheresis for primary prevention of stroke, and whose clinical course was complicated by chylothorax which was successfully treated with a pleuroperitoneal shunt.

## 1. Introduction

Chylothorax arises when lymphatic fluid (chyle) accumulates in the pleural cavity because of leakage from lymphatic vessels. The incidence of chylothorax in children is unknown. Although a rare cause of pleural effusion in most children, it is the most common form of pleural effusion in neonates [1]. It is commonly caused by direct injury to the thoracic duct after surgery, or the infiltration of the lymphatic system secondary to malignant diseases. Central vein thrombosis causes backpressure in the thoracic duct return, and chyle subsequently leaks into the pleural cavity. Central vein thrombosis as a cause of chylothorax is uncommon, and in a few cases in the literature was related to thrombotic complications of central venous access devices (CVAD). Superior vena cava (SVC) occlusion-induced chylothorax has been described in adult sickle cell disease (SCD) in a setting of chronic indwelling CVAD [2]. There are limited reports on chylothorax induced by central venous thrombosis secondary to chronic CVAD in children with SCD.

## 2. Case Report

**Figure 1:**
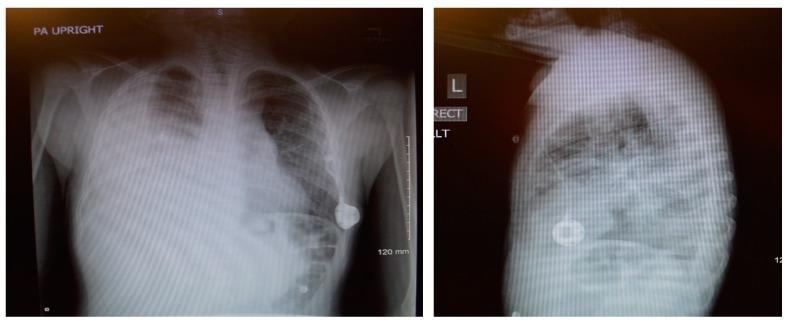
Chest X-rays demonstrating undrained right chylothorax.

An 8-year-old male patient with a history of SCD maintained on long term erythrocytapheresis (LTE) for primary prevention of stroke due to a history of increased transcranial Doppler velocities presented with several days of fatigue, decreased oral intake, chest pain, and palpitations. Imaging studies showed a large right-sided pleural effusion with underlying atelectasis or consolidation (Figure 1). A chest tube was inserted for drainage of the pleural fluid, which continued to have significant output for several weeks. The pleural fluid had features consistent with chylous effusion, including elevated triglycerides, positive for chylomicrons, and milky appearance.

**Figure 2:**
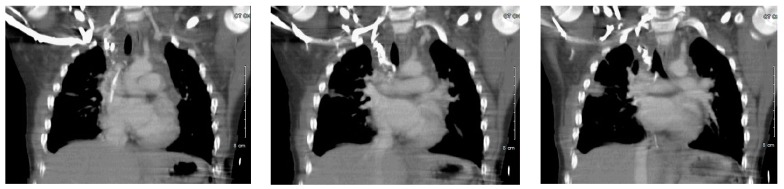
Chest computed tomography (CT) (intravenous (IV) contrast) scan demonstrating inability to fully visualize the superior vena cava (SVC) in total, and no opacification of the right internal jugular (IJ). Images from left to right demonstrate CT slices in an anterior to posterior fashion, respectively.

**Figure 3:**
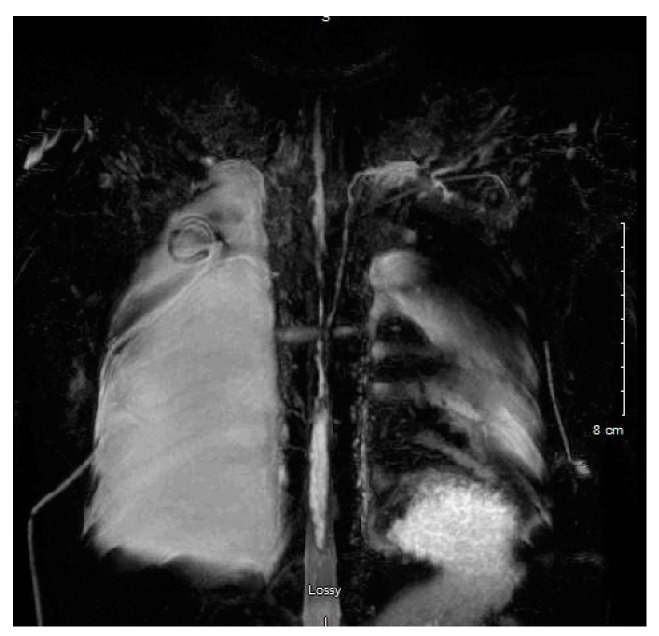
Magnetic resonance image (MRI) showed two separate lymphatic ducts draining the lymph from cisterna chyli to the right and left subclavian veins.

Enhanced computed tomography (CT) venogram was performed (Figure 2); followed two weeks later by conventional venogram, which showed right internal jugular (IJ) vein thrombus with SVC extension. The patient did not respond to dietary therapy, consisting of a low fat diet, showing little improvement in the pleural drainage. An interventional radiology lymphangiogram was inconclusive because the contrast was very slow moving. A thoracic magnetic resonance image (MRI) showed two separate lymphatic ducts draining the lymph from cisterna chyli to the right and left subclavian veins (Figure 3). This represented a normal congenital variant with the (left) thoracic duct smaller in caliber. There were two draining sites at the terminal part of the (left) thoracic duct, with the accessory draining site inferior and posterior to the main opening. The terminal part of the main (right) thoracic duct at the level of confluence of the right IJ and right subclavian vein could not be delineated.

A thrombophilia workup looking for traditional factors revealed the following:

**Test****Result**Prothrombin time (PT)11.2 sInternational normalized ratio (INR)1.1Partial thromboplastin time (PTT)30.8 sFragment D-Dimer (fibrin degradation product)1.8 mg/L (high)Homocysteine4.8 μmol/L (low)Prothrombin Gene MutationNegativeFactor V LeidenHeterozygous for mutationAntithrombin III123.3%Protein C activity104%Protein S activity-Functional77%Antiphospholipid antibody panelNegativeAnticardiolipin antibody panelNegativeLipoprotein A57 mg/dL (high)Methylenetetrahydrofolate reductase (MTHFR) mutationNegative

The etiology of his thrombus was likely from the presence of a central line used for LTE in the setting of a known hypercoagulable state of SCD and heterozygous factor V Leiden mutation. He was therefore placed on long term anticoagulant therapy using low molecular weight heparin. Aspirin was also added. The pleural drainage remained significant so he was made “nothing by mouth” (NPO) and given total parenteral nutrition for 3 weeks. A bilateral upper extremity venogram 2 weeks after presentation showed a persistent clot in the SVC and multiple collaterals. The tip of the CVAD was in the right brachiocephalic vein. He was then started on octreotide and the dose was increased but provided no improvement in the chest tube output. He subsequently underwent vascular surgery procedures including thrombectomy—which was unsuccessful as the vascular surgeon was unable to cross the subclavian lesion with both a Kumpe catheter and a soft angle Glide wire—followed by balloon angioplasty of the SVC and left innominate vein. The SVC and left innominate vein showed resolution of stenosis, however there was still occlusion of the subclavian vein with several collaterals. At the same time, his CVAD was removed. He was discharged with the chest tube in place.

**Figure 4:**
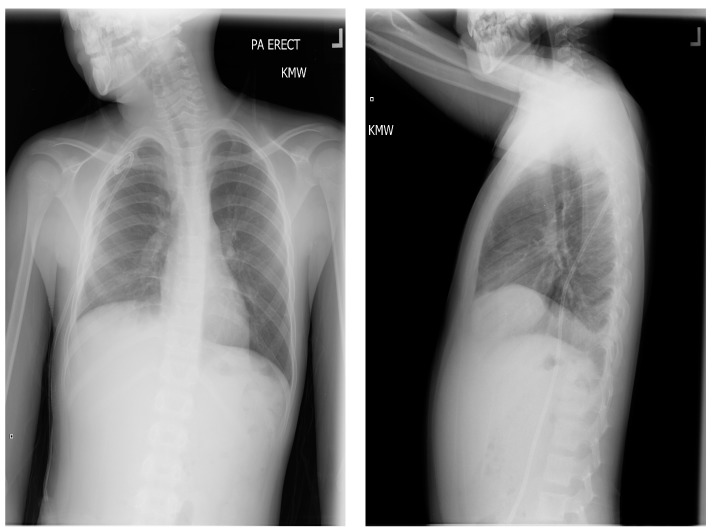
Pre-operative chest X-rays obtained about 1 month after initial presentation, with pigtail chest tube and persistent right chylous effusion (based on imaging and persistent chylous output from chest tube).

**Figure 5:**
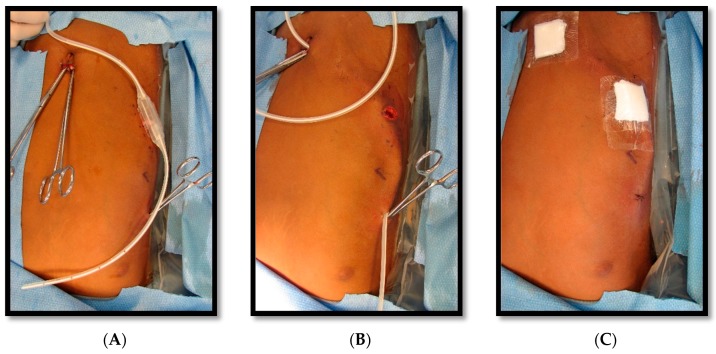
Operative photos of the insertion of the reversed pleuroperitoneal shunt. Right abdomen and chest shown; head is at top. From left to right: (**A**) shunt tubing laid over body, with instruments accessing right chest via axilla, and abdomen via umbilicus; (**B**) shunt tunneled under skin; and (**C**) final result with bandages.

**Figure 6:**
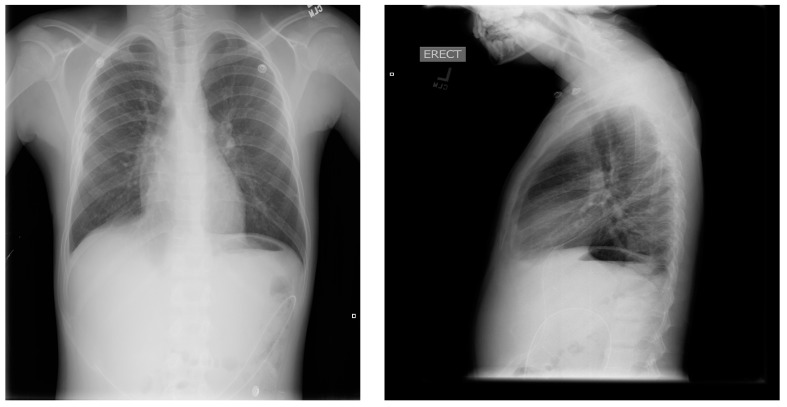
Post-operative chest X-rays, showing reversed pleuroperitoneal shunt in right chest and abdomen.

He returned to the hospital 2 weeks later due to persistent drainage, erythema, and pain around the chest tube site, for which he was treated with 10-day course of clindamycin. Following discharge, he returned 2 weeks later for a reversed pleuroperitoneal (PP) shunt placement with perioperative transfusion, and measures to prevent acute chest syndrome. The shunt was a single valve, 15.5 French, Denver (Beckton, Dickinson and Company, Franklin Lakes, NJ, USA) PP shunt, used in this case in a reverse fashion such that the valve opened from pleural cavity to the abdominal cavity (Figure 4, Figure 5 and Figure 6). (This particular PP shunt is typically designed to drain malignant abdominal ascites into the chest cavity).

**Figure 7:**
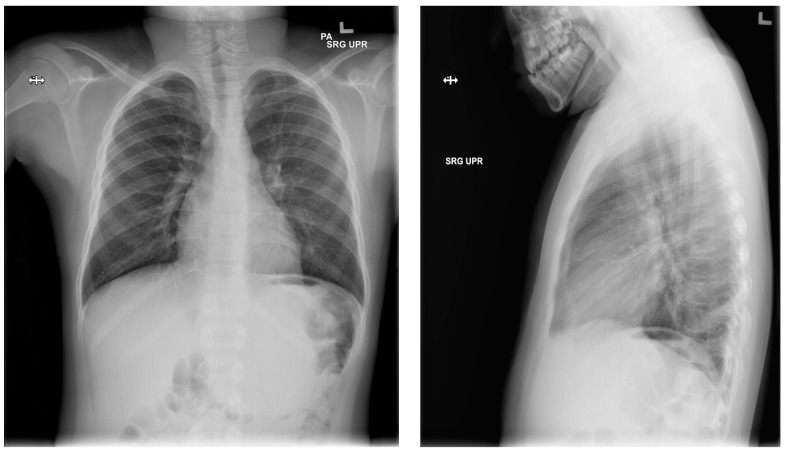
Most recent chest X-ray, several months after removal of shunt.

The pressure gradient necessary to open the valve is 3 cm of water, but this can be overcome by compressing the bulb which houses the one-way valve, forcing flow from chest to abdomen. This pressure gradient is noted on the packing insert for the shunt and is outlined within the instruction manual for the Denver shunt on the manufacturer, Beckton, Dickinson, and Company’s (Franklin Lakes, NJ, USA) website. The patient continued with monthly simple transfusions, and hydroxyurea was instituted. Following the PP shunt placement, he required one hospitalization for re-accumulated chylothorax and had a thoracostomy tube placed. Significant drainage was felt to be secondary to the obstruction of the PP catheter and to non-adherence regarding compressing the shunt reservoir bulb daily in order to prevent obstruction of its one-way valve. The PP catheter was cleared by pressure on the shunt bulb, which was continued at home a few times daily. Repeat imaging with Duplex ultrasound showed no change in the thrombus, consistent with fibrotic changes. The PP shunt was removed about 6 months after placement, and he has remained free of pleural effusion (Figure 7). The decision to remove the shunt was based on an asymptomatic period of about 6 months, parent comfort level, and the surgeon’s past experience with PP shunts. He has continued to be managed with hydroxyurea, with dose escalation to the maximally tolerated dose, and he has transitioned off transfusions.

## 3. Discussion

Chlyothorax is not a commonly known complication related to pediatric SCD. There are no published studies demonstrating a correlation between them. However, central venous access device-related venous thromboembolism (VTE) in SCD patients appears to be high in both adult and pediatric populations. Studies including adult SCD patients have demonstrated that 12–30% of CVADs are complicated by VTE, with an incidence ranging from 0.18 to 0.99 VTE events per 1000 catheter days [3]. Pediatric-only studies have also shown high incidence of thrombotic complications (15–16% per CVAD) in SCD patients [3]. In contrast, studies investigating clinically symptomatic CVAD-related VTE in cancer patients report VTE incidence ranges of 0.3–28.3% per catheter in adult patients, with substantially lower VTE rates in pediatric populations (0–3.1% in most studies) [4]. The high CVAD–VTE rates in SCD pediatric patients may be due to the significant underlying hypercoagulability in SCD, or differences in CVAD-related factors such as type of catheter or duration of CVC implantation [3]. Furthermore, the observed high incidence of CVAD-related VTE in pediatric SCD patients is seen in other chronic inheritable diseases such as cystic fibrosis, and may reflect increased coagulability in the setting of chronic illness and inflammation [5].

Literature review reveals the use of a PP shunt in cases of chronic chylothorax, refractory chylothorax, and lymphatic dysplasia/anomaly [6]. However, there is little information on the use of a PP shunt in cases of chylothorax in SCD. In pediatrics, the most common uses of a PP shunt has been in congenital chylothorax due to a lymphatic anomaly or chylothorax as a post-operative complication, most commonly from repair of congenital heart disease [7]. As early as 1999, a review of 15 cases was done in children with PP shunts with exteriorized pump chambers and the researchers found the mean age at time of placement was 2.1 years (with an age range of 0.1–11.5 years), the most common indication was refractory chylothorax after repair of congenital heart disease, the mean chylothorax duration before shunt placement was 76 days (with a range of 5–810 days) and shunts were in place for an average of 104 days (with a range of 12–365 days) [8]. Another study in 1999 reviewed the use of PP shunts in 7 infants aged 10–66 days for refractory chylothorax, in which 4 were congenital and 3 were post-surgical (cardiothoracic, SVC thrombosis removal, and diaphragmatic hernia repair) in nature [9].

In our patient, the cause of his persistent chylothorax was likely related to thrombus resulting from the presence of a CVAD which had migrated from the SVC to the right brachiocephalic vein, secondary to a prolonged duration from its implantation. Another contributing factor to his right IJ thrombus was the underlying hypercoagulability associated with SCD. Further research may need to be done in determining the frequency of monitoring the location of a CVAD in children who may experience migration of the catheter related to the anatomical changes in a growing child.
